# Phase 1, open-label study of MEDI-547 in patients with relapsed or refractory solid tumors

**DOI:** 10.1007/s10637-012-9801-2

**Published:** 2012-02-28

**Authors:** Christina M. Annunziata, Elise C. Kohn, Patricia LoRusso, Nicole D. Houston, Robert L. Coleman, Manuela Buzoianu, Gabriel Robbie, Robert Lechleider

**Affiliations:** 1 Medical Oncology Branch, Center for Cancer Research, National Cancer Institute, National Institutes of Health, 10 Center Drive, Room 12 N226, Bethesda, MD 20892-1906 USA; 2 Barbara Ann Karmanos Cancer Institute, Wayne State University, Detroit, MI 48201 USA; 3 Department of Gynecologic Oncology, University of Texas MD Anderson Cancer Center, Houston, TX 77030 USA; 4 MedImmune, LLC, Gaithersburg, MD 20878 USA; 5 Human Genome Sciences, Inc, Rockville, MD 20850 USA

**Keywords:** MEDI-547, EphA2, Cancer therapy, Clinical trial, Relapsed/refractory solid tumors

## Abstract

*Background* Targeting the cell-surface receptor EphA2, which is highly expressed in some solid tumors, is a novel approach for cancer therapy. We aimed to evaluate the safety profile, maximum tolerated dose (MTD), pharmacokinetics, and antitumor activity of MEDI-547, an antibody drug conjugate composed of the cytotoxic drug auristatin (toxin) linked to a human anti-EphA2 monoclonal antibody (1C1), in patients with solid tumors relapsed/refractory to standard therapy. *Methods* In this phase 1, open-label study with planned dose-escalation and dose-expansion cohorts, patients received a 1-h intravenous infusion of MEDI-547 (0.08 mg/kg) every 3 weeks. *Results* Six patients received 0.08 mg/kg; all discontinued treatment. Dose escalation was not pursued. The study was stopped before cohort 2 enrollment due to treatment-related bleeding and coagulation events (hemorrhage-related, *n* = 3; epistaxis, *n* = 2). Therefore, lower doses were not explored and an MTD could not be selected. The most frequently reported treatment-related adverse events (AEs) were increased liver enzymes, decreased hemoglobin, decreased appetite, and epistaxis. Three patients (50%) experienced treatment-related serious AEs, including conjunctival hemorrhage, pain (led to study drug discontinuation), liver disorder, and hemorrhage. Best response included progressive disease (*n* = 5; 83.3%) and stable disease (*n* = 1; 16.7%). Minimal or no dissociation of toxin from 1C1 conjugate occurred in the blood. Serum MEDI-547 concentrations decreased rapidly, ~70% by 3 days post-dose. No accumulation of MEDI-547 was observed at 0.08 mg/kg upon administration of a second dose 3 weeks following dose 1. *Conclusions* The safety profile of MEDI-547 does not support further clinical investigation in patients with advanced solid tumors.

## Introduction

Ephrin type-A receptor 2 (EphA2), a member of the Eph family of receptor tyrosine kinases, is involved in developmental cell-to-cell interactions and cell migration processes, including angiogenesis and neural crest cell migration [1, 2]. These events occur through the interaction of the Eph receptors with their ligands, the ephrins, that are anchored to the membrane of adjacent cells. While EphA2 is expressed at relatively low levels in normal adult tissues [1, 3], it is overexpressed in a number of carcinomas, including ovarian, endometrial, and cervical cancers; melanoma; and gliomas [4–16]. EphA2 overexpression in several types of cancers has been correlated with poor patient outcome [17–20]. Collectively, these findings suggest that EphA2 may be an ideal tumor target for cancer therapy. In addition to serving as a direct target, EphA2 could be used as a cancer-related protein for antibody-targeting therapy, particularly antibody-drug conjugate (ADC)-based therapies, in which chemical toxins or radioligands are covalently added to the antibody structure [21].

MEDI-547 is an ADC composed of a human immunoglobulin (Ig) G1 monoclonal antibody directed against EphA2 (known as 1C1) and conjugated on cysteine residues to an auristatin derivative linker-toxin maleimidocaproyl-monomethyl auristatin phenylalanine (mcMMAF) [22]. The antibody has no antitumor effects when unconjugated [22]; thus, it was intended to be used to deliver highly toxic chemotherapy directly to EphA2-expressing cancer cells. Auristatin is a microtubule inhibitor that binds to the vinca alkaloid-binding domain, and has been shown to exhibit potent anticancer effects in preclinical and clinical studies [11, 23, 24]. MEDI-547 binds human, cynomolgus monkey, mouse, and rat EphA2 with similar binding affinities via the highly conserved extracellular domain [7, 22] (MedImmune, LLC, data on file). Upon internalization of MEDI-547, cysteine (cys)-mcMMAF is released from 1C1 by lysosomal degradation of the antibody component, inducing cell cycle arrest at the G2 – M border, microtubule disruption, and apoptotic cell death [7, 22].

A primary concern for any new targeted cancer therapy is the range and sensitivity of potentially susceptible tissues, both normal and malignant. A Good Laboratory Practice-compliant tissue cross-reactivity study of MEDI-547 conducted on a full panel of normal human tissues from 3 donors demonstrated rare, weak staining of the epithelium of tonsilar crypts and esophageal mucosa, and occasional, weak cytoplasmic staining of placental trophoblastic epithelium. All other tissues were negative [25]. In tumor xenograft studies in nude mice, MEDI-547 inhibited the growth of EphA2-expressing tumors with no obvious adverse effects [7, 15, 22]. The intravenous dose at which antitumor activity was observed in mice was 1–3 mg/kg [22]. Based on preclinical efficacy studies, the anticipated efficacious dose in humans was 1.2 mg/kg once every 3 weeks (q3wks) or 0.405 mg/kg every week (qwk).

Based on these and other preclinical results, a phase 1, open-label first-in-human study was conducted to investigate the safety profile, tolerability, and maximum tolerated dose (MTD) of MEDI-547 in patients with relapsed or refractory solid tumors. Secondary objectives included evaluation of pharmacokinetics, immunogenicity, and antitumor activity.

## Methods

### Study population

Patients at least 18 years of age and with a life expectancy >16 weeks were eligible for enrollment into the study if they had a histologically confirmed malignant solid tumor thought to be associated with increased expression of EphA2 (endometrial, breast, ovarian, prostate, non-small cell lung, colon, esophageal, gastric, and bladder cancers, renal cell carcinoma, melanoma) [4–15], relapsed or refractory to standard therapy, and an Eastern Cooperative Oncology Group (ECOG) performance status score of 0–2. Laboratory criteria included absolute neutrophil count ≥1,500/μL, platelet count ≥100,000/μL, hemoglobin >10.0 g/dL, serum creatinine ≤1.5 times the upper limit of normal (ULN) or calculated creatinine clearance >50 mL/min, serum bilirubin ≤2 times the ULN, alkaline phosphatase <3 times the ULN, and aspartate aminotransferase (AST) and alanine aminotransferase (ALT) ≤3 times the ULN. Prothrombin time, partial thromboplastin time, and international normalized ratio were required to be within normal institutional limits.

Patients with active infectious disease, known brain metastases, or a serious illness thought to interfere with the protocol were excluded from the study, as were those who were treated with other investigational drugs or had participated in another clinical trial within 30 days before the start of therapy or concomitantly with this trial. Patients with a known secondary malignancy that required therapy, abnormal hematologic values, impaired renal or liver function were also excluded, as were those patients who had any evidence of hematemesis, melena, hematochezia, ≥ grade 2 hemoptysis, or gross hematuria or who were on anticoagulant therapy for thromboembolic disorders or prophylactic reasons.

Concomitant medications from 30 days prior to the first dose through 30 days after the last dose of MEDI-547 were minimized to those medications necessary for management of active comorbid diseases and symptoms related to cancer burden or treatment of side effects. The following medications were considered exclusionary: (1) investigational agents for anticancer treatment or supportive care; and (2) any antitumor therapy other than MEDI-547, such as cytostatic and/or cytotoxic drugs, hormonal therapy, radiation therapy, immunotherapy, or any biological response modifiers.

The protocol was reviewed and approved by the Institutional Review Board of the two participating sites. Written informed consent was obtained from each patient before study entry.

### Study design

Patients were to receive MEDI-547 as a 1-h intravenous (IV) infusion once q3wks or qwk for 3 consecutive weeks until unacceptable toxicity, progressive disease, initiation of alternative anticancer therapy, or other reasons for withdrawal. The study included a standard 3 + 3 dose-escalation design. The starting dose of 0.08 mg/kg was selected as 1-tenth the highest non-severely toxic dose (5 mg/kg) in the rat, which was determined to be the most sensitive species in toxicity studies. Subsequent cohort dose levels were to be increased by 50% to 10 mg/kg or until determination of the MTD. Enrollment into the weekly dosing cohorts would begin after the 0.27 mg/kg dose level in the q3wks schedule was considered to have no safety concerns based on dose-escalation rules.

### Safety profile and tolerability assessments

Adverse events (AEs) were graded according to the National Cancer Institute Common Terminology Criteria for Adverse Events (CTCAE) version 3.0. Physical examination, vital signs, electrocardiogram, performance status, laboratory evaluations including coagulation studies, and AE and serious AE (SAE) monitoring were used to assess safety. For non-hematologic toxicities, dose-limiting toxicity (DLT) was defined as any ≥grade 3 toxicity except grade 3 or 4 nausea and vomiting for <24 h and grade 3 non-hematologic laboratory abnormalities that resolve to grade 1 within 7 days. For hematologic toxicities, DLT was defined as grade 4 neutropenia for >7 days, grade 4 febrile neutropenia (or grade 3 requiring antibiotics), or grade 4 platelets (<25,000/μL) at any time. DLTs were assessed within 21 days after treatment initiation with MEDI-547 for cycle 1. The MTD was to be defined on the basis of DLT observed during the first cycle of treatment. If 1 of 3 patients experienced a DLT in the first cycle, an additional 3 patients were to be enrolled. If ≥2 patients at the same dose level experienced a DLT, the MTD would be exceeded and a total of 6 patients would be treated at the preceding dose. If ≤1 of 6 patients experienced a DLT, then this dose level would be the MTD. Once the MTD was determined, the study planned to suspend enrollment into higher dose cohorts, and expand the MTD patient cohort.

### Immunogenicity evaluation

Blood samples were collected before infusion of MEDI-547 and at the end of treatment for determination of anti-MEDI-547 antibody levels. The immunogenicity assay for measuring anti-MEDI-547 antibodies was built upon a bridging format using electrochemiluminescence (ECL) technology platform developed by Meso Scale Discovery (MSD). Briefly, biotinylated MEDI-547 and ruthenylated MEDI-547 were allowed to form a complex with the analyte (anti-MEDI-547 antibodies) contained in the serum samples during an overnight incubation. The anti-drug antibodies (ADA) bridged complexes were captured by the streptavidin-coated MSD plate and detected by MSD Sector Imager.

### Pharmacokinetic evaluation

Blood samples were collected for measurement of MEDI-547 antibody-drug conjugate, MEDI-547 ADC + 1C1 (total 1C1, unconjugated and conjugated), cys-mcMMAF (the linear form of the toxin component), and cyclic cys-mcMMAF (the cyclic form of the toxin component) concentrations following administration of MEDI-547 ADC. Two enzyme-linked immunosorbent assays (ELISA) were used to measure the serum concentrations of the IgG-cys-mcMMAF conjugate (ADC) and the IgG (total 1C1). The lower limit of quantification (LLOQ) was 0.5 μg/mL for both ADC and 1C1. A validated liquid chromatography-mass spectrometry (LC/MS) assay was used to determine the plasma levels of cys-mcMMAF and cyclic cys-mcMMAF. Briefly, the analysis of cyc-mcMMAF and cyc-mcMMAF cyclic was conducted on an HPLC system interfaced with a mass spectrometer, using turbospray ionization in the positive ion mode. The analyte and internal standard (m + 7 cyc-mcMMAF) were detected by multiple reactions. The LLOQ was 2 ng/mL for both cys-mcMMAF and cyclic cys-mcMMAF. Serum and plasma concentrations were summarized. Pharmacokinetic parameters were not estimated due to limited concentration data at low dose (0.08 mg/kg by 1-h infusion q3wks).

### Clinical activity assessment

Tumor assessments were performed at screening and approximately every 6 weeks (2 cycles) after the first dose of MEDI-547. Disease response was assessed by tumor measurements, evaluated according to Response Evaluation Criteria in Solid Tumors version 1.0 [26] and defined as a complete response, partial response, stable disease, or progressive disease.

### Statistical analyses

Safety, efficacy, and pharmacokinetic characteristics were analyzed in an exploratory and descriptive manner. All patients who received at least one dose of MEDI-547 were included in the efficacy and safety analyses.

## Results

### Patient population

The study was conducted at two clinical sites in the United States between August 4, 2009, and January 26, 2010. A total of 6 patients were enrolled, all in the 0.08 mg/kg q3wks treatment group. All patients discontinued treatment due to progressive disease (*n* = 4), AEs (*n* = 1), or planning to initiate a different treatment (*n* = 1). This last patient was also assessed as having progressive disease. Four patients discontinued the study due to withdrawal of consent and 2 patients discontinued for other reasons (notification of study closure and planning to initiate other treatment). All patients were women, most were Caucasian, and the median age was 63 years (range: 49–75). All patients previously had chemotherapy; most previously had surgery and had experienced radiation, hormonal, or another type of cancer therapy (Table 1). The median number of prior chemotherapy regimens was 3 (range: 1–8). The primary tumor types included endometrial (*n* = 3), breast (*n* = 1), colon (*n* = 1), and ovarian (*n* = 1) cancer. All patients had metastatic cancer at study entry.

**Table 1:** Previous treatments

Therapy	No. (%) of patients (*N* = 6)
Biologic	3^a^ (50.0)
Chemotherapy	6^a^ (100.0)
Radiation	3^b,c^ (50.0)
Surgery	5 (83.3)
Hormonal	2^a^ (33.3)
Other	1^a^ (16.7)

^a^100% were in a neoadjuvant/adjuvant setting

^b^66.7% were in a neoadjuvant/adjuvant setting

^c^Radiation therapy was palliative care in 1 patient

### Safety profile and tolerability

All 6 patients were exposed to MEDI-547 at a dose of 0.08 mg/kg once every 3 weeks. Four patients received 1 treatment cycle and 2 patients received 2 treatment cycles. All patients experienced at least one AE, with a total of 70 events reported. The most common (≥3 patients) AEs reported were increased ALT and decreased serum albumin concentrations (50% each). Five patients (83.3%) experienced at least one treatment-related AE, with a total of 36 such events reported (Table 2). The majority of treatment-related AEs were grade 1 (23 AEs) and 2 (12 AEs) in severity; one was classified as grade 3 (pain). Four patients (66.7%) experienced 5 serious AEs (SAEs): conjunctival hemorrhage, constipation, pain, liver disorder, and hemorrhage. With the exception of constipation, these SAEs, reported in 3 patients (50%), were considered by the investigator to be treatment-related; 1 SAE (pain) led to discontinuation of the study drug in 1 patient. No deaths occurred during the study.

**Table 2:** Treatment-related adverse events^a^

Adverse event	No. (%) of patients (*N* = 6)
ALT increased	3 (50.0)
AST increased	2 (33.3)
Hemoglobin decreased	2 (33.3)
Decreased appetite	2 (33.3)
Epistaxis	2 (33.3)
Angina pectoris	1 (16.7)
Conjuctival hemorrhage	1 (16.7)
Eye pain	1 (16.7)
Mouth hemorrhage	1 (16.7)
Nausea	1 (16.7)
Fatigue	1 (16.7)
Pain	1 (16.7)
Liver disorder	1 (16.7)
Blood albumin decreased	1 (16.7)
Blood alkaline phosphatase increased	1 (16.7)
Blood glucose increased	1 (16.7)
Blood magnesium increased	1 (16.7)
Blood sodium increased	1 (16.7)
Platelet count decreased	1 (16.7)
WBC count decreased	1 (16.7)
Headache	1 (16.7)
Depression	1 (16.7)
Dyspnea	1 (16.7)
Pleural effusion	1 (16.7)
Hemorrhage	1 (16.7)

*ALT* alanine aminotransferase; *AST* aspartate aminotransferase; *WBC* white blood cell

^a^Related is defined as possibly, probably, or definitely related to study drug

Five of 6 patients (83.3%) experienced bleeding and coagulation events. Three patients had hemorrhage-related events, and 2 patients reported epistaxis. Three patients had bleeding/coagulation AEs that were also SAEs. The occurrence of these bleeding and coagulation events in the first dose cohort was the reason this study was closed early (Table 3).

**Table 3:** Bleeding- and coagulation-related adverse events

Adverse event	Cycle (C)/Day (D)	Severity	Relationship to MEDI-547	SAE
Pain^a^	C1/D22	Grade 3	Possible	Yes
Liver Disorder^a^	C1/D22	Grade 2	Possible	Yes
Conjunctival Hemorrhage	C1/D8	Grade 1	Possible	Yes
Epistaxis	C1/D3	Grade 1	Possible	No
Mouth Hemorrhage	C1/D4	Grade 1	Possible	No
Epistaxis^b^	C1/D7	Grade 1	Possible	No
Hemorrhage^b^	C1/D9	Grade 2	Possible	Yes

*SAE* serious adverse event

^a^SAEs in the same patient

^b^SAEs in the same patient

### Immunogenicity

All six patients had immunogenicity assessments conducted at the end of treatment, all of which were negative for anti-MEDI-547 antibodies. One of these patients tested positive (titer = 20) at cycle 1, day 1 pre-dose, at a level just above the LLOQ (titer = 10).

### Pharmacokinetics

Following IV administration of MEDI-547 ADC at 0.08 mg/kg by 1-h infusion every 3 weeks, serum MEDI-547 ADC concentrations were generally similar to MEDI-547 ADC + 1C1, indicating minimal or no dissociation of toxin from 1C1 conjugate in the blood. Plasma concentrations for cys-mcMMAF and cyclic cys-mcMMAF were undetectable (LLOQ = 2 ng/mL) at all time points in all patients. At 0.5 h after the end of infusion, serum concentrations of MEDI-547 ADC and MEDI-547 ADC + 1C1 in all 6 patients were measurable, and the mean values were 2.140 and 2.058 μg/mL, respectively. Serum concentrations of MEDI-547 ADC and MEDI-547 ADC + 1C1 decreased approximately 70% by 3 days post-dose, and the mean values were 0.670 and 0.728 μg/mL, respectively. Serum concentrations for both ADC and ADC + 1C1 were below detection limit (LLOQ = 0.5 μg/mL) 7 days post-dose.

Two patients received a second dose of MEDI-547 3 weeks later with mean serum concentration of 2.175 μg/mL for both MEDI-547 ADC and MEDI-547 ADC + 1C1 at 0.5 h after the end of infusion, indicating no accumulation at this dose level and with a 3-week dosing interval.

### Clinical activity

Five patients had an overall response of progressive disease and 1 patient had an overall response of stable disease. No complete or partial tumor responses were observed.

## Discussion

This was a phase 1, open-label study that was intended to assess a dose of IV MEDI-547 q3wks in patients with solid tumors relapsed or refractory to standard therapy. Six patients were accrued with 4 receiving only one cycle of treatment. The study was stopped before enrollment of dose-escalation cohort 2 due to bleeding and coagulation events that occurred in 5 of 6 patients. The perceived overall risk of a serious bleeding event at higher doses was felt to be sufficiently high that continued clinical evaluation was deemed unsafe. Based on animal studies, we expected that antitumor activity would begin to occur at 1.2 mg/kg q3wks (dose level 8). Thus, the likelihood of reaching an efficacious dose without unacceptable toxicity was low because of the types and severity of toxicity that were observed at the starting dose, and the magnitude of the difference between starting and expected efficacious doses.

Bleeding and coagulation events were consistent with the disease profile and may have been associated with disease progression, but MEDI-547 was also a likely cause of these events. The patient with an SAE of hemorrhage was admitted to the emergency room after reporting hemoptysis; she had numerous large pulmonary metastases. However, her hemoptysis stopped with discontinuation of MEDI-547, despite continued growth of the pulmonary metastases.

The appearance of clotting abnormalities was not unexpected based on the preclinical toxicology findings, but the potential severity was unknown in a clinical setting. The bleeding and coagulation events observed in humans showed some similarities to those evident in rats and monkeys [25]. In all three species, increased activated partial thromboplastin time, increased fibrinogen/fibrin degradation product, and increased fibrin D-dimer were reported. Monkeys had red/blood discharge from the nose, mouth, gums and/or anus, whereas two patients experienced epistaxis and one patient experienced an oral cavity hemorrhage. Preclinical toxicology studies in the monkey identified disseminated intravascular coagulation (DIC) as the DLT. The events observed in humans were considered to be consistent with the preclinical findings, in particular to the observation of DIC.

The dose used in the clinical study was 10-fold lower than the highest non-severely toxic dose predicted based on extrapolations from rat studies (MedImmune, LLC, data on file), and no evidence of drug accumulation after the administration of a second dose was apparent.

Although auristatin is an effective cytotoxic agent for the treatment of many types of cancers, it exposes patients to a considerable risk for peripheral neuropathies, as do other therapies using microtubule-stabilizing agents [27, 28]. Peripheral neuropathy and associated adverse events were reported in 36% of patients in a recent phase 1 study that treated CD30-positive lymphoma patients with brentuximab vedotin, another ADC containing auristatin [29]. Most adverse events were reported for patients who were treated with 1.8 or 2.7 mg/kg. The adverse events observed in patients treated with MEDI-547, however, were of a different class than those observed in patients treated with brentuximab vedotin (hematologic events vs. neuropathies) and were observed following treatment with lower doses. Rash, possibly due to the expression of glycoprotein non-metastatic melanoma protein B (GPNMB) in healthy skin, was the most common toxicity reported after treatment with glembatumumab, another ADC containing monomethyl auristatin E that is being evaluated for the treatment of GPNMB-expressing breast cancers and melanomas [30]. Neutropenia, another predictable AE associated with auristatin, was also reported as a grade 3 DLT in clinical studies of glembatumumab [11].

Nonspecific toxicity from auristatin is unlikely to explain the observed events in the present study, because serum MEDI-547 concentrations were generally similar to MEDI-547 + 1C1 concentrations, indicating minimal or no dissociation of toxin from the antibody in the blood. Furthermore, cys-mcMMAF and cyclic cys-mcMMAF were not detected in the serum of patients. Based on these data and the experiences with brentuximab vedotin and glembatumumab, the antibody component of the ADC (1C1) probably underlies the MEDI-547 toxicity observed in the present study.

Previous studies did not support the possibility that MEDI-547 targeted EphA2 in normal tissues. Preclinical evaluation found no reactivity of MEDI-547 in target organs of the rat or monkey (MedImmune, LLC, data on file) [25]. MEDI-547 did bind non-specifically to most target tissues at the highest doses administered, and toxicities were most commonly hyperplasias, increased mitotic figures, and necroses. Non-specific binding was more likely to have occurred in these preclinical models because the top dose used in the toxicity study was approximately 12-fold greater than the efficacious dose required for a rat tumor model, and 60-fold higher than the starting dose in the clinical study (MedImmune, LLC, data on file) [25]. Another study also suggested that potential targeting of MEDI-547 to normal tissue was minimal, with preferential distribution to tumors and no accumulation in normal mouse or rat tissues [31]. Targeting of MEDI-547 to normal tissues must be reassessed, however, in light of the results of this clinical study. Previous preclinical analyses of human tissue cross-reactivity demonstrated weak membrane staining of the epithelium of tonsilar crypts and esophageal mucosa, and weak cytoplasmic staining of placental trophoblastic epithelium (MedImmune, LLC, data on file). The weak tissue reactivity was considered rare and not significant enough to warrant cessation of drug development in the preclinical stage. It is now important to reconsider these findings, as these tissues may represent targets for MEDI-547 underlying the AEs experienced by the patients in this study.

Despite the results of this study, EphA2 remains a potential target for cancer therapeutics, because it is overexpressed in a number of cancer types, and this is correlated with poor patient outcome [17–20]. A wide range of therapeutic strategies are under development using monoclonal antibodies, immunoconjugates, small-molecule antagonists, adenovirus vectors, immunotherapy, RNA interference, vaccination, and nanoparticles [32]. For example, dasatinib, an orally active small-molecule tyrosine kinase inhibitor that has potent inhibitory activity against EphA2 [33], is being evaluated in clinical studies for the treatment of squamous cell carcinoma (ClinicalTrials.gov identifier: NCT00563290), in combination with chemotherapy for endometrial cancer (ClinicalTrials.gov identifier: NCT01440998), in combination with radiotherapy for glioblastoma (ClinicalTrials.gov identifier: NCT00895960), and in combination with bevacizumab for patients with solid tumors (ClinicalTrials.gov identifier: NCT00792545). Another approach using a combination therapy of EphA2 small interfering RNA (siRNA) with the chemotherapeutic drug paclitaxel was more effective in inhibiting growth of HeyA8 or SKOV3 orthotopic ovarian tumors in mice than was treatment with the control siRNA and paclitaxel [34]. In yet another approach, nanoshells targeting EphA2 were used to kill EphA2-overexpressing PC-3 cells but not EphA2-deficient human dermal fibroblast cells [35]. The results observed so far with some of these therapeutic approaches support EphA2 as a cancer target. In addition, the favorable clinical data reported using ADCs such as brentuximab vedotin and glembatumumab also support the use of ADCs. Thus, our study results do not preclude ADC cancer therapies targeting EphA2 as yet. Before additional studies are considered, however, the cause of the AEs observed in this study must be further explored. Specifically, the MEDI-547 target in epithelial tissues included in the preclinical human tissue cross-reactivity study should be identified. If EphA2 is expressed in these tissues, this would eliminate it as a potential target for ADC cancer therapies. Alternatively, it is possible that MEDI-547 may cross react with another protein, perhaps another Eph family member, again preventing further study of MEDI-547. An alternate antibody could then be considered for use as an ADC cancer therapy.
